# The Effect of Internet-Based Cognitive Behavioral Therapy on Major Depressive Disorder: Randomized Controlled Trial

**DOI:** 10.2196/42786

**Published:** 2023-09-22

**Authors:** Ziyi Lin, Lu Cheng, Xue Han, Hongqiong Wang, Yuhua Liao, Lan Guo, Jingman Shi, Beifang Fan, Kayla M Teopiz, Muhammad Youshay Jawad, Huimin Zhang, Yan Chen, Ciyong Lu, Roger S McIntyre

**Affiliations:** 1 Department of Medical Statistics and Epidemiology School of Public Health Sun Yat-sen University Guangzhou China; 2 Guangdong Provincial Key Laboratory of Food Nutrition and Health Sun Yat-sen University Guangzhou China; 3 Guangdong Engineering Technology Research Center of Nutrition Translation Guangzhou China; 4 Department of Psychiatry Shenzhen Nanshan Center for Chronic Disease Control Shenzhen China; 5 Brain and Cognition Discovery Foundation Toronto, ON Canada; 6 Mood Disorders Psychopharmacology Unit University Health Network Toronto, ON Canada; 7 Department of Pharmacology University of Toronto Toronto, ON Canada; 8 Department of Psychiatry University of Toronto Toronto, ON Canada

**Keywords:** depressive symptoms, major depressive disorder, internet-based cognitive behavioral therapy, self-efficacy, stigma, social function, health-related quality of life, mental health services

## Abstract

**Background:**

Many people living with major depressive disorder (MDD) in China do not receive treatment owing to a lack of mental health services, along with significant stigma toward mental illness. Internet-based cognitive behavioral therapy (ICBT) has been proposed to increase access to mental health care for people with MDD.

**Objective:**

The aims of this study were to (1) evaluate the efficacy of ICBT for depressive symptoms in patients with MDD; (2) evaluate the effect of ICBT on anxiety symptoms, nonspecific psychological distress, general self-efficacy, depression stigma, social function, and health-related quality of life (HRQoL); and (3) explore the acceptability of and satisfaction with the ICBT program among participants.

**Methods:**

Patients with MDD were enrolled and randomized to the ICBT group or the waiting-list control (WLC) group. The ICBT group received ICBT delivered through a WeChat mini-program with general support by nonspecialists. Participants in the 2 groups were self-evaluated online at baseline and posttreatment for changes in the primary outcome (ie, depressive symptoms) and secondary outcomes (ie, anxiety symptoms, nonspecific psychological distress, general self-efficacy, depression stigma, social functional impairment, and HRQoL). Changes in outcomes were measured by changes in overall scores on respective scales, and response and remission rates were calculated based on depressive symptoms. The acceptability of and satisfaction with the ICBT program were measured by treatment adherence and participants’ feelings (ie, modules seriously completed, perceived benefit, and satisfaction).

**Results:**

We included 40 patients who were randomly assigned to the ICBT group and 44 who were assigned to the WLC group. Compared with the WLC group, the ICBT group had fewer depressive symptoms, fewer anxiety symptoms, less nonspecific psychological distress, and greater general self-efficacy. Moreover, the ICBT group had higher response (18/31, 58%) and remission rates (17/31, 55%). The adherence rate in the ICBT group was 78% (31/40), and the majority of participants who completed all ICBT modules were satisfied with the ICBT program.

**Conclusions:**

ICBT demonstrated greater improvements in depressive symptoms, anxiety symptoms, nonspecific psychological distress, and general self-efficacy among selected patients with MDD in comparison with the findings in waiting-list controls. The ICBT program in this study had good acceptability and satisfaction among participants.

**Trial Registration:**

Chinese Clinical Trial Registry (ChiCTR2100046425); https://tinyurl.com/bdcrj4zv

## Introduction

Major depressive disorder (MDD) is a chronic debilitating condition associated with impaired social function [1], decreased quality of life [2], higher risk of suicide [3], and higher mortality [4]. The lifetime prevalence of MDD in China is 3.4%, and the 12-month prevalence is 2.1%, indicating that approximately 50 million patients with MDD need access to effective treatment [5]. However, the China Mental Health Survey (CMHS) reported that only 67 (11.6%) of 655 participants with MDD received any treatment for their depressive symptoms, and only 11 (0.8%) met the criteria of minimally adequate treatment [6], which is far lower than that in high-income countries (22.4%) or in upper-middle-income countries (11.4%) [7]. Barriers to effective treatment include scarce mental health services, lack of trained mental health specialists, and stigma associated with mental disorders [7-9]. Digital psychological services have been considered as important measures for addressing unmet mental health treatment needs, as they may increase access to mental health services outside of a clinical setting at lower costs [10,11].

The most widely used form of digital psychological intervention for MDD is internet-based cognitive behavioral therapy (ICBT). ICBT is based on the principles of cognitive behavioral therapy (CBT), which aims to evaluate and modify a patient’s dysfunctional thoughts and rectify maladaptive behaviors [12]. Indeed, the American Psychiatric Association Practice Guidelines [13] and Chinese Guideline for Prevention and Treatment of Depression [14] recommend CBT as a first-line treatment for MDD. CBT has been reported to not only have a considerable effect in the acute treatment of MDD [15], but also reduce the risk of relapse or recurrence [16].

Many randomized controlled trials (RCTs) have been carried out to examine the effectiveness of ICBT for alleviating depressive symptoms, and they have reported varied results with small [17] to large [18,19] effect sizes, as well as no effect size [20]. In China, ICBT is also gradually being used to improve mental health. Some studies implemented ICBT in Chinese patients before and after surgery to reduce anxiety and depressive symptoms [21,22], and some implemented ICBT to alleviate the anxiety and distress of people during the COVID-19 epidemic [23-25]. Ying et al [26] implemented an ICBT intervention for Chinese adults with subthreshold depression (obtained a score of ≥16 on the Center for Epidemiological Studies Depression Scale and did not meet the criteria for a major depressive episode) and found that ICBT was effective in reducing depressive symptoms in the study population and that improvements in outcomes were sustained at a 6-month follow-up. Only 1 ICBT study focused on Chinese outpatients with MDD [27], but the ICBT program for the intervention was the Chinese translated version of MoodGYM, which was developed by the Centre for Mental Health Research at The Australian National University [28]. Cultural adaptability might influence the effect of such an intervention, and the convenience of implementing ICBT interventions needs to be further improved. More studies are needed to evaluate the effect of ICBT in Chinese patients with MDD.

ICBT can be classified as guided or unguided. Guided ICBT has been defined as ICBT that involves therapeutic support delivered by specialists, while unguided ICBT has been defined as ICBT that is delivered automatically and allows technical support but not support related to the therapeutic content [29]. Unguided ICBT is more affordable and operative [30], but guided ICBT is more effective [29,31], which is generally thought to be associated with better treatment adherence [32,33]. However, the number of available specialist providers to deliver therapeutic support or guidance is limited, and nonspecialist health workers, like teachers, nurses, or lay health workers, may therefore have an important role to play in delivering support [34].

This study aimed to (1) evaluate the efficacy of ICBT for depressive symptoms in patients with MDD; (2) evaluate the effect of ICBT on patients’ anxiety symptoms, nonspecific psychological distress, general self-efficacy, stigma associated with depression, social function, and health-related quality of life (HRQoL); and (3) explore the acceptability of and satisfaction with the ICBT program applied among participants, so as to provide a reference for the subsequent promotion of digital psychological services.

## Methods

### Study Design

This study was a 2-armed and nonblinded RCT focused on the effect of ICBT in patients with MDD. Participants were randomized into the following 2 groups: (1) ICBT group (ICBT with general support by nonspecialists) and (2) waiting-list control (WLC) group (treatment as usual [35]). The outcomes of both groups were measured at baseline and posttreatment.

### Ethical Considerations

This trial was approved by the Ethics Review Committee of Shenzhen Nanshan Center for Chronic Disease Control (approval number: ll20210012) and was preregistered in the Chinese Clinical Trail Registry (registration number: ChiCTR2100046425). All participants provided informed consent. All personal information in the study data has been deidentified to protect the privacy of the participants.

### Participants and Procedure

All participants were recruited from the Department of Depressive Disorder of Shenzhen Kangning Hospital and the Department of Psychiatry of Shenzhen Nanshan Center for Chronic Disease Control between August and December 2021.

The inclusion criteria of this study were as follows: (1) age 18-60 years; (2) meeting the criteria for MDD in the Diagnostic and Statistical Manual of Mental Disorders, Fifth Edition (DSM-V); (3) a score of more than 5 on the Patient Health Questionnaire-9 (PHQ-9), indicating the presence of at least mild depressive symptoms [36]; (4) if taking antidepressants, being on a stable dose for at least 4 weeks; and (5) access to an internet-connected smartphone. The exclusion criteria were as follows: (1) history of neurological diseases; (2) moderate or high risk of suicide according to section C of the Mini-International Neuropsychiatric Interview (MINI) [37]; (3) currently undergoing physiotherapy or psychotherapy treatment; (4) pregnancy or breastfeeding; (5) severe physical disease; (6) psychosis or bipolar disorder; and (7) substance use disorder.

Psychiatrists visited outpatients to identify potential participants for our study. Trained researchers performed structured diagnostic interviews, which consisted of the MINI, with potential participants to assess eligibility for enrollment in our study. Then, researchers obtained informed consent from all eligible participants and guided them through the online baseline assessment. Random assignment of all participants was performed by a computer randomization program before recruitment. Participants were randomly assigned to the ICBT group or the WLC group. It was not possible for participants or study researchers to be blinded to the treatment allocation owing to the nature of the intervention.

### Intervention

#### ICBT Group

The research team developed an ICBT intervention delivered through a Chinese WeChat mini-program called *Morning Mood*. This program, which consists of 7 treatment modules corresponding to conventional face-to-face CBT courses of treatment, aims to teach users how to manage their depressive symptoms using CBT skills, including cognitive restructuring, problem solving, and relapse prevention. Multimedia Appendix 1 shows an outline of the treatment content and homework of each module. Participants in the ICBT group were required to complete 1 module per week. A new module was opened automatically on the 7th day after participants completed the previous module, so as to ensure that they would not complete multiple modules in a short time, which could influence the effect of the intervention.

The ICBT group also received general support from trained nonspecialists (ie, any type of health worker, like a nurse, lay health worker, or medical social worker, who is not a specialist in mental health) via telephone or WeChat to remind and encourage them to complete the ICBT modules on time and to manage any technical problems with the program. We conducted systematic training for nonspecialists before the start of this study, including the intervention process and purpose, the use and background management of the ICBT program, the identification and reporting of adverse events, and the communication skills with participants. The principle of general support was not to provide any form of psychotherapy. The ICBT program also included a module of symptom self-assessment so that users could monitor and adjust their depressive or anxiety symptoms by themselves. A week after finishing all modules, participants were asked to complete the online posttreatment assessment.

#### WLC Group

Participants in the WLC group maintained their treatment as usual without any additional intervention in the first 8 weeks after enrollment, and they were asked to complete the posttreatment assessment by the end of the 8th week. They were provided access to the ICBT program after finishing the online posttreatment assessment.

### Measures

#### Demographic and Health-Related Information and Adverse Experience Measures

Participant demographic and health-related data, such as nationality, occupation, marital status, anamnesis (including hypertension, diabetes, heart disease, stroke, thyroid disease, cancer, and infectious disease), antidepressant use, and mental health services received during this study, were recorded.

The Child Trauma Questionnaire (CTQ) was used to measure participants’ 5 different forms of childhood trauma [38]. The cutoff values of physical neglect, physical abuse, emotional neglect, emotional abuse, and sexual abuse are 10, 10, 15, 13, and 8 points, respectively [39]. As long as any subscale score reaches the cutoff value, it is determined that the participant has experienced childhood maltreatment. Its Cronbach α was .62 in this study.

The household dysfunction questionnaire was adapted from the Centers for Disease Control and Prevention, and Kaiser Permanente adverse childhood experiences study [40]. It consists of 9 items to investigate whether an individual had experiences such as parents who had divorced, death of one or both parents, and economic difficulties before the age of 16 years (see Multimedia Appendix 2 for further details). Selecting “yes” for any item indicates that the individual has had household dysfunction.

The list of adverse life events (a 16-item list) was derived from the List of Threatening Experiences (LTE) (high test-retest reliability; kappa range=0.61-0.87) [41] and Life Event Scale (LES) (satisfactory test-retest reliability; *r*=0.61-0.74) [42,43]. It lists 16 kinds of adverse life events, including but not limited to serious illness, economy, law, marriage, and work (see Multimedia Appendix 3 for further details). Participants were categorized according to the number of recent adverse life events (0, 1-3, or ≥4).

#### Primary Outcome Measures

The PHQ-9, a self-report inventory that has sufficient psychometric properties [44], has been widely used in studies on ICBT to assess depressive symptoms [45,46]. Its Cronbach α was .88 in this study. Response was defined as a 50% or more reduction in the baseline PHQ-9 score, and remission was defined as a PHQ-9 score of less than 5 at the posttreatment assessment [47].

#### Secondary Outcome Measures

The Generalized Anxiety Disorder-7 Scale (GAD-7) is a 4-point scale measuring the severity of anxiety symptoms. It has demonstrated satisfactory reliability and validity [48]. Its Cronbach α was .90 in this study.

The Kessler 10-item Psychological Distress scale (K-10) was used to evaluate nonspecific psychological distress over the past 4 weeks [49]. It is rated on a 5-point scale, and a higher score represents a higher distress level. Its Cronbach α was .92 in this study.

The General Self-Efficacy Scale (GSES), a 10-item scale with good reliability and validity, was used to measure one’s own belief of whether he or she has the ability to complete a certain behavior [50]. It is rated on a 4-point scale, and a typical item is as follows: “Thanks to my resourcefulness. I can handle unforeseen situations.” A higher total score indicates greater self-confidence. Its Cronbach α was .92 in this study.

Participants’ attitudes toward depression were assessed using the Depression Stigma Scale (DSS), which comprises 2 subscales: personal and perceived depression stigma [51]. The personal depression stigma subscale measures the degree to which participants personally agree with statements related to depression (eg, “depression is a sign of personal weakness”). The perceived depression stigma subscale corresponds to the personal depression stigma subscale, which measures participants’ perceptions of most people’s views on depression (eg, “most people believe that depression is a sign of personal weakness”). Each item was answered on a 5-point Likert scale, and a higher score represents stronger stigma. Its Cronbach α was .85 in this study.

The Sheehan Disability Scale (SDS) is a well-validated measurement and was used to assess social functional impairment in patients with MDD [52]. It is a composite of three 10-point scale response items that aim to assess the level of participants’ impairment due to depressive symptoms with regard to their work or school work, social life or leisure activities, and family life or family responsibilities. Its Cronbach α was .90 in this study.

The Short Form 6-Dimension (SF-6D) was used to measure HRQoL. It contains 6 questions corresponding to 6 dimensions (physical function, role limitations, social function, pain, mental health, and vitality), with 4 to 6 levels each, and produces a health utility value that ranks health states on a scale of 0.00 (death) to 1.00 (full health) [53]. Its Cronbach α was .80 in this study.

#### Acceptability of and Satisfaction With the ICBT Program

The completion status of participants in the ICBT group was used to describe the acceptability of the ICBT program. In addition, those who finished all modules were asked to rate their performance and feelings about the ICBT program, including the number of modules they carefully completed, the perceived benefit, their satisfaction, and whether they would recommend the ICBT program to their family or friends with depressive symptoms (see Multimedia Appendix 4 for the detailed questionnaire).

### Statistical Analysis

Baseline characteristics were compared between 2 groups using *t* tests for continuous variables and chi-square tests or Fisher exact probabilities for categorical variables. A multiple linear regression model was used for each outcome at posttreatment. Each model took the outcome as the dependent variable and the grouping as the independent variable (the WLC group as reference), and included age, gender, smoking, drinking, antidepressant use, childhood maltreatment, household dysfunction, recent adverse life events, mental health services received during this study, and respective baseline outcome measure scores as covariates [54-57]. In addition, each outcome measure score at posttreatment was compared between all ICBT module completers and noncompleters in the ICBT group, using covariance analysis, and the respective baseline outcome measure scores were included as covariates. Response and remission rates were calculated based on depressive symptoms and were compared between the ICBT and WLC groups using chi-square tests. A significance level (*P* value) of <.05 (2-tailed) was used for all analyses. All statistical analyses were performed using SPSS version 26.0 (IBM Corp).

Because clinical trials involving ICBT interventions for patients with depressive symptoms have been reported to have poor adherence [29], the effect of ICBT estimated by an intention-to-treat analysis may be underestimated [58,59]. We aimed to evaluate the effect of actually completing the full course of an ICBT intervention. Therefore, this study was mainly based on per-protocol analysis. The per-protocol set (PPS) included participants who finished all modules in the ICBT group and who completed the posttreatment assessment in the WLC group. To comply with the intention-to-treat principle, this study contacted participants who did not complete all modules in the ICBT group for posttreatment assessment, and regarded the analysis based on the full analysis set (FAS) as sensitivity analysis. The FAS included participants who completed the posttreatment assessment in the 2 groups.

## Results

### Participant Characteristics

**Figure 1:**
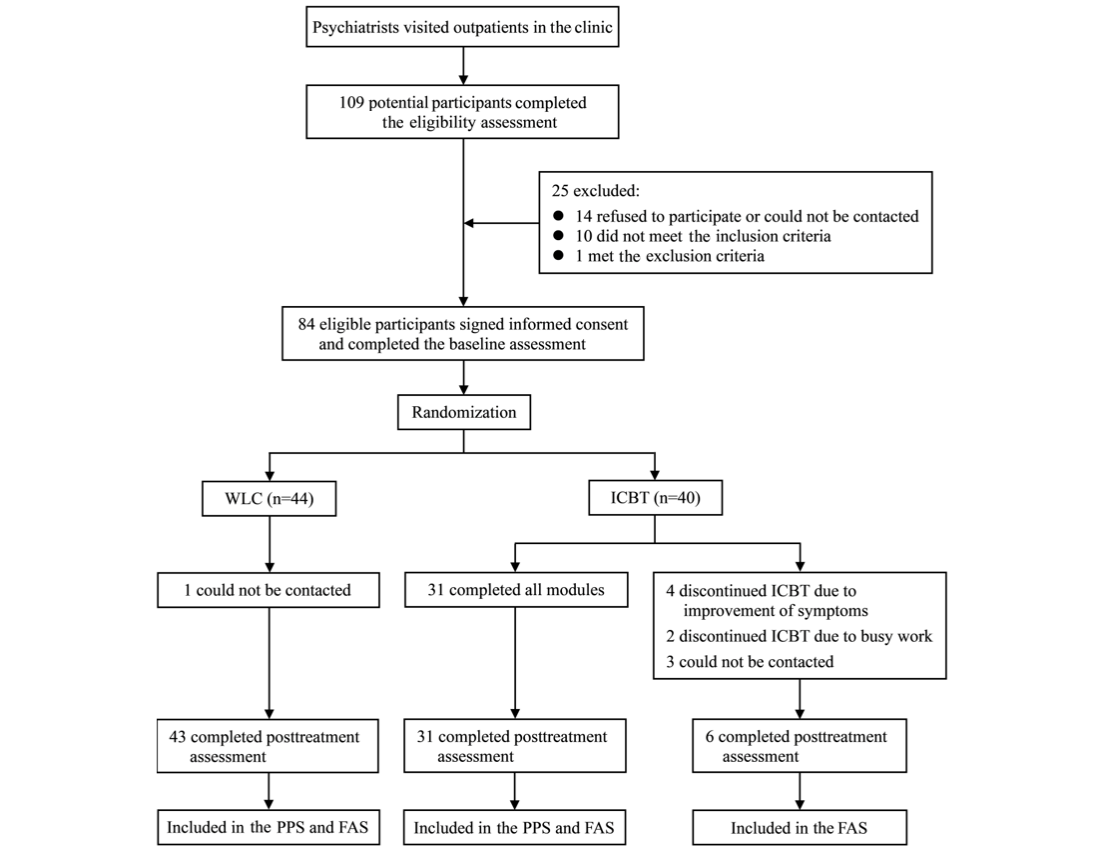
Flow of participants through randomization, assessment, and analysis. FAS: full analysis set; ICBT: internet-based cognitive behavioral therapy; PPS: per-protocol set; WLC: waiting-list control.

The study enrolled 84 patients with MDD, and of these, 40 were randomly assigned to the ICBT group and 44 were assigned to the WLC group. Figure 1 provides details of participant randomization and assessment. The baseline characteristics of the participants are presented in Table 1. Most participants were female (62/84, 74%) and of Han nationality (80/84, 95%), and the mean participant age was 30.82 (SD 8.14) years. Moreover, the majority had an undergraduate degree (60/84, 71%) and were employed (63/84, 75%). Participants reported moderate depressive symptoms, with a mean score of 12.87 (SD 5.48). The mean scores of the GAD-7, K-10, GSES, personal depression stigma subscale, perceived depression stigma subscale, SDS, and SF-6D were 9.71 (SD 5.03), 28.64 (SD 8.18), 19.95 (SD 5.71), 22.43 (SD 6.00), 30.22 (SD 7.01), 12.99 (SD 7.01), and 0.67 (SD 0.12), respectively. There were no differences between the ICBT and WLC groups in terms of demographic and health-related information, adverse experiences, and outcome measures at baseline.

**Table 1:** Baseline characteristics of the participants.

Characteristic		Total (N=84)	ICBT^a^ group (n=40)	WLC^b^ group (n=44)	*P* value^c^
Age (years), mean (SD)		30.82 (8.14)	31.48 (8.27)	30.23 (8.08)	.49
**Sex, n (%)**					.45
	Male	22 (26)	12 (30)	10 (23)	
	Female	62 (74)	28 (70)	34 (77)	
**Nationality, n (%)**					.68
	Han nationality	80 (95)	39 (98)	41 (93)	
	Other nationality	4 (5)	1 (2)	3 (7)	
**Education status, n (%)**					.12
	High school or below	11 (13)	8 (20)	3 (7)	
	Undergraduate	60 (71)	28 (70)	32 (73)	
	Master’s degree or above	13 (16)	4 (10)	9 (20)	
**Occupation, n (%)**					.20
	Student	7 (8)	1 (2)	6 (14)	
	Employed	63 (75)	31 (78)	32 (72)	
	Unemployed	14 (17)	8 (20)	6 (14)	
**Marital status, n (%)**					.22
	Never married	46 (55)	18 (45)	28 (64)	
	Married	34 (40)	19 (47)	15 (34)	
	Divorced/widowed	4 (5)	3 (8)	1 (2)	
**Smoking, n (%)**					.30
	Yes	30 (36)	12 (30)	18 (41)	
	No	54 (64)	28 (70)	26 (59)	
**Drinking, n (%)**					.27
	Yes	71 (85)	32 (80)	39 (89)	
	No	13 (15)	8 (20)	5 (11)	
**Anamnesis^d^, n (%)**					.92
	Yes	32 (38)	15 (38)	17 (39)	
	No	52 (62)	25 (62)	27 (61)	
**Antidepressant use, n (%)**					.71
	Yes	48 (57)	22 (55)	26 (59)	
	No	36 (43)	18 (45)	18 (41)	
**Childhood maltreatment, n (%)**					.60
	Yes	59 (70)	27 (68)	32 (73)	
	No	25 (30)	13 (32)	12 (27)	
**Household dysfunction, n (%)**					.45
	Yes	66 (79)	30 (75)	36 (82)	
	No	18 (21)	10 (25)	8 (18)	
**Recent adverse life events, n (%)**					.24
	0	7 (8)	2 (5)	5 (11)	
	1-3	19 (23)	26 (65)	32 (73)	
	≥4	58 (69)	12 (30)	7 (16)	
PHQ-9^e^ score, mean (SD)		12.87 (5.48)	13.10 (5.96)	12.66 (5.07)	.72
GAD-7^f^ score, mean (SD)		9.71 (5.03)	9.85 (5.21)	9.59 (4.93)	.82
K-10^g^ score, mean (SD)		28.64 (8.18)	27.70 (7.64)	29.50 (8.65)	.32
GSES^h^ score, mean (SD)		19.95 (5.71)	21.05 (5.33)	18.95 (5.92)	.09
Personal depression stigma subscale score, mean (SD)		22.43 (6.00)	22.33 (5.77)	22.52 (6.27)	.89
Perceived depression stigma subscale score, mean (SD)		30.22 (7.01)	30.03 (7.12)	30.39 (6.99)	.82
SDS^i^ score, mean (SD)		12.99 (7.01)	13.28 (7.80)	12.73 (6.29)	.72
SF-6D^j^ score, mean (SD)		0.67 (0.12)	0.67 (0.13)	0.66 (0.11)	.77

^a^ICBT: internet-based cognitive behavioral therapy.

^b^WLC: waiting-list control.

^c^Baseline characteristics were compared between the 2 groups using *t* tests for continuous variables and chi-square tests or Fisher exact probabilities for categorical variables.

^d^Anamnesis includes hypertension, diabetes, heart disease, stroke, thyroid disease, cancer, and infectious disease.

^e^PHQ-9: Patient Health Questionnaire-9.

^f^GAD-7: Generalized Anxiety Disorder-7 Scale.

^g^K-10: Kessler 10-item Psychological Distress scale.

^h^GSES: General Self-Efficacy Scale.

^i^SDS: Sheehan Disability Scale.

^j^SF-6D: Short Form 6-Dimension.

### Primary Outcomes

**Figure 2:**
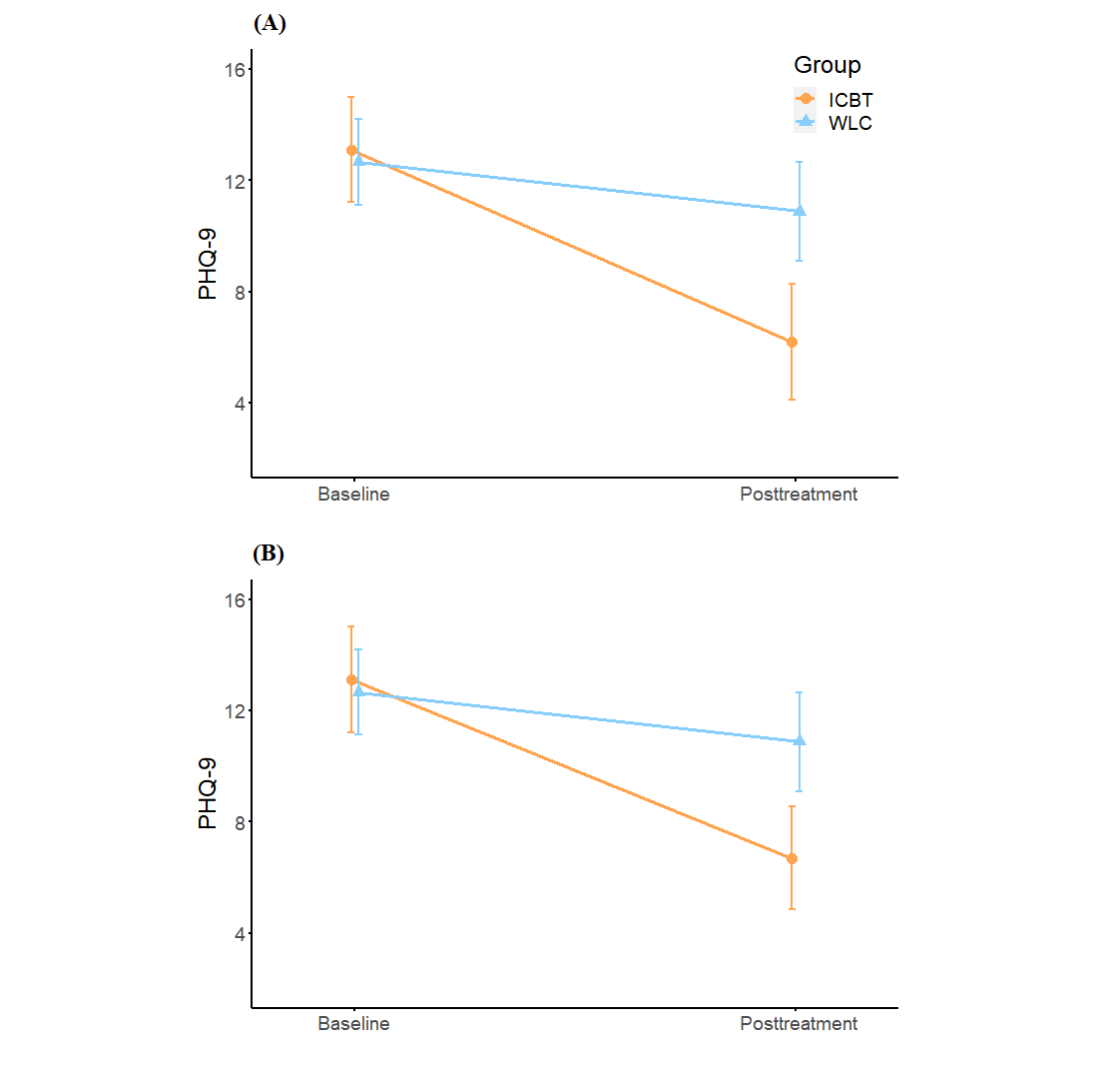
Changes in the PHQ-9 scores over time in the (A) PPS and (B) FAS. FAS: full analysis set; ICBT: internet-based cognitive behavioral therapy; PHQ-9: Patient Health Questionnaire-9; PPS: per-protocol set; WLC: waiting-list control.

Figure 2 illustrates changes in PHQ-9 scores over time. Table 2 presents the mean scores of outcome measures at posttreatment and the results of the multiple linear regression. After adjusting for confounding factors, the PHQ-9 score was lower in the ICBT group than in the WLC group at posttreatment assessment (PPS analysis: *β*=−4.827, 95% CI −7.033 to −2.621; FAS analysis: *β*=−4.662, 95% CI −6.738 to −2.587). However, there was no statistically significant difference in the PHQ-9 score between completers and noncompleters in the ICBT group at the posttreatment assessment (*F*_1_=1.752; *P*=.19; see Table 3 for further details). Furthermore, based on the PPS, we used covariance analysis to compare the PHQ-9 score of participants who had experienced a local COVID-19 outbreak and those who had not in the 2 groups at the posttreatment assessment, and found that there was no difference within the ICBT group (*F*=0.295; *P*=.59) and the WLC group (*F*=0.099; *P*=.76; see Multimedia Appendix 5 for further details).

**Table 2:** Results of outcome measures at the posttreatment assessment.

Analysis set and outcome measure		ICBT^a^ group, mean (SD)	WLC^b^ group, mean (SD)	*β*, value (95% CI)^c^	*P* value^c^
**PPS^d^**					
	PHQ-9^e^ score	6.19 (5.68)	10.88 (5.80)	−4.827 (−7.033 to −2.621)	<.001
	GAD-7^f^ score	5.32 (5.29)	7.77 (5.61)	−2.784 (−4.897 to −0.670)	.01
	K-10^g^ score	20.77 (11.83)	26.42 (9.33)	−4.688 (−8.693 to −0.682)	.02
	GSES^h^ score	24.19 (5.44)	20.30 (5.84)	3.262 (0.755 to 5.770)	.01
	Personal depression stigma subscale score	22.10 (8.01)	23.21 (6.38)	−0.936 (−4.207 to 2.336)	.57
	Perceived depression stigma subscale score	28.77 (9.26)	30.88 (7.39)	−2.207 (−6.082 to 1.669)	.26
	SDS^i^ score	9.61 (7.46)	10.49 (7.56)	−0.855 (−3.757 to 2.046)	.56
	SF-6D^j^ score	0.72 (0.10)	0.70 (0.12)	0.021 (−0.029 to 0.071)	.41
**FAS^k^**					
	PHQ-9 score	6.68 (5.55)	10.88 (5.80)	−4.662 (−6.738 to −2.587)	<.001
	GAD-7 score	5.65 (5.54)	7.77 (5.61)	−2.907 (−4.944 to −0.871)	.006
	K-10 score	21.35 (11.52)	26.42 (9.33)	−4.827 (−8.761 to −0.894)	.02
	GSES score	24.00 (5.36)	20.30 (5.84)	2.889 (0.494 to 5.284)	.02
	Personal depression stigma subscale score	22.46 (7.61)	23.21 (6.38)	−0.600 (−3.664 to 2.465)	.70
	Perceived depression stigma subscale score	28.54 (9.03)	30.88 (7.39)	−1.880 (−5.599 to 1.839)	.32
	SDS score	9.54 (7.33)	10.49 (7.56)	−0.966 (−3.726 to 1.793)	.49
	SF-6D score	0.72 (0.11)	0.70 (0.12)	0.024 (−0.025 to 0.072)	.33

^a^ICBT: internet-based cognitive behavioral therapy.

^b^WLC: waiting-list control.

^c^A multiple linear regression model was used for each outcome at posttreatment. Each model took the outcome as the dependent variable and the grouping as the independent variable (WLC group as reference), and age, gender, smoking, drinking, antidepressant use, childhood maltreatment, household dysfunction, recent adverse life events, mental health services received during this study, and respective baseline outcome measure scores were included as covariates. The regression coefficient *β* of the grouping and its 95% CI were calculated, and *P* values of *t* tests for the regression coefficient were obtained.

^d^PPS: per-protocol set.

^e^PHQ-9: Patient Health Questionnaire-9.

^f^GAD-7: Generalized Anxiety Disorder-7 Scale.

^g^K-10: Kessler 10-item Psychological Distress scale.

^h^GSES: General Self-Efficacy Scale.

^i^SDS: Sheehan Disability Scale.

^j^SF-6D: Short Form 6-Dimension.

^k^FAS: full analysis set.

**Table 3:** Adjusted outcome measure scores of all module completers and noncompleters in the internet-based cognitive behavioral therapy group at the posttreatment assessment.

Outcome measure	Completers^a^ (n=31), mean (SD)	Noncompleters^a^ (n=6), mean (SD)	*F* test (*df*)	*P* value
PHQ-9^b^ score	6.24 (0.82)	8.94 (1.87)	1.752 (1)	.19
GAD-7^c^ score	5.46 (0.84)	6.64 (1.92)	0.315 (1)	.58
K-10^d^ score	20.85 (1.94)	23.97 (4.41)	0.419 (1)	.52
GSES^e^ score	24.39 (0.91)	22.00 (2.10)	1.075 (1)	.31
Personal depression stigma subscale score	21.99 (1.40)	24.57 (3.14)	0.562 (1)	.46
Perceived depression stigma subscale score	28.96 (1.72)	27.70 (4.00)	0.082 (1)	.78
SDS^f^ score	9.47 (1.13)	9.89 (2.57)	0.022 (1)	.88
SF-6D^g^ score	0.72 (0.02)	0.70 (0.04)	0.290 (1)	.59

^a^Adjusted mean score of each outcome measure at posttreatment assessment using covariance analysis with the respective baseline outcome measure scores as covariates.

^b^PHQ-9: Patient Health Questionnaire-9.

^c^GAD-7: Generalized Anxiety Disorder-7 Scale.

^d^K-10: Kessler 10-item Psychological Distress scale.

^e^GSES: General Self-Efficacy Scale.

^f^SDS: Sheehan Disability Scale.

^g^SF-6D: Short Form 6-Dimension.

### Secondary Outcomes

According to the PPS analysis, participants in the ICBT group had less anxiety symptoms, nonspecific psychological distress, and greater general self-efficacy than those in the WLC group (all *P*<.05; see Table 2 for further details). There were no significant differences in the scores of the personal and perceived depression stigma subscales, SDS, or SF-6D between the groups. The FAS analysis reported similar results. In addition, Table 3 depicts the comparison between completers and noncompleters in the ICBT group at posttreatment, using covariance analysis. There were no significant differences in secondary outcomes between completers and noncompleters. Moreover, there were no significant differences in the scores of the GAD-7 or K-10 between participants who had experienced the COVID-19 outbreak and those who had not within both the ICBT and WLC groups (Multimedia Appendix 5).

### Response and Remission of Depressive Symptoms

Table 4 shows the response and remission rates of the participants’ depressive symptoms. The results of PPS analyses showed that the ICBT group had a response rate of 58% (18/31) and a remission rate of 55% (17/31), while the WLC group had a response rate of 16% (7/43) and a remission rate of 9% (4/43). The results of FAS analyses showed that the response and remission rates of the ICBT group decreased slightly (19/37, 51% and 17/37, 46%, respectively). The ICBT group demonstrated a significantly greater response to treatment in comparison to the WLC group, with significantly greater rates of remission (all *P*<.05; see Table 4 for further details).

**Table 4:** The response and remission rates of participants based on depressive symptoms.

Analysis set and outcome measure		ICBT^a^ group, % (n/N)	WLC^b^ group, % (n/N)	*χ*^2^ (*df*)	*P* value
**PPS^c^**					
	Response rate^d^	58 (18/31)	16 (7/43)	14.060 (1)	<.001
	Remission rate^e^	55 (17/31)	9 (4/43)	18.377 (1)	<.001
**FAS^f^**					
	Response rate	51 (19/37)	16 (7/43)	11.151 (1)	.001
	Remission rate	46 (17/37)	9 (4/43)	13.794 (1)	<.001

^a^ICBT: internet-based cognitive behavioral therapy.

^b^WLC: waiting-list control.

^c^PPS: per-protocol set.

^d^Number of participants who had a 50% or more reduction in the baseline Patient Health Questionnaire-9 (PHQ-9) score at the posttreatment assessment divided by the number of analyzed participants.

^e^Number of participants who had a PHQ-9 score of less than 5 at the posttreatment assessment divided by the number of analyzed participants.

^f^FAS: full analysis set.

### Acceptability of and Satisfaction With the ICBT Program

Among the 40 participants in the ICBT group, 31 (78%) completed all modules, 1 (2%) completed 6 modules, 1 (2%) completed 5 modules, 2 (5%) completed 3 modules, 2 (5%) completed 2 modules, and 3 (8%) completed only 1 module (the first module). Table 5 shows the results of questions about performance and feelings about the ICBT program among participants who completed all modules in the ICBT group. Among the 31 participants who completed all modules, 24 (78%) reported that they had seriously completed half or more of the treatment modules, 13 (42%) perceived great or enormous benefit from ICBT, 22 (71%) were satisfied or very satisfied with the ICBT program, and 28 (90%) said that they would recommend this ICBT program to their family or friends with depressive symptoms.

**Table 5:** Results of questions about performance and feelings about the internet-based cognitive behavioral therapy program among participants who completed all modules.

Item	Modules seriously completed^a^ (N=31), n (%)	Perceived benefit^b^ (N=31), n (%)	Satisfaction^c^ (N=31), n (%)
1	0 (0)	0 (0)	0 (0)
2	7 (22)	6 (19)	0 (0)
3	12 (39)	12 (39)	9 (29)
4	8 (26)	9 (29)	15 (48)
5	4 (13)	4 (13)	7 (23)

^a^The items were as follows: 1, almost none; 2, fraction; 3, half; 4, majority; 5, all.

^b^The items were as follows: 1, none at all; 2, a little; 3, moderate; 4, great; 5, enormous.

^c^The items were as follows: 1, very dissatisfied; 2, dissatisfied; 3, average; 4, satisfied; 5, very satisfied.

## Discussion

### Principal Findings

This study delivered a 7-module ICBT intervention with general support by nonspecialists to patients with MDD, and compared the findings to those in a WLC group (treatment as usual). The intervention support in most ICBT studies was provided by professional therapists (eg, individual therapist chat sessions [60], regular supervision, and feedback via email [61]), which limits the promotion of ICBT. Our study increased the practicability of implementing ICBT by considering nonspecialists as support personnel. Moreover, most previous studies set a definite assessment time regardless of whether participants completed the full ICBT course [17-19]. However, our study aimed to evaluate the effect of ICBT on patients with MDD after completing all ICBT modules. Similar to the finding in other ICBT studies involving patients with MDD, the majority of participants in this study were female. However, compared with Australian studies [45,62], the participants in this study were younger, more participants were undergraduates, more participants were unmarried, and more participants were taking antidepressants. The results of this study indicate that individuals in the ICBT group experienced a significant reduction in depressive symptoms and higher remission rates in comparison to individuals in the WLC group at the posttreatment assessment. Our results demonstrate that the efficacy of routine treatment combined with ICBT for MDD was better than treatment as usual. The results of FAS analyses (as sensitivity analyses) provided similar conclusions, which means that our results are robust. Johansson et al [63] delivered an 8-week ICBT program in a routine psychiatric setting and concluded that ICBT appears to be an effective treatment for depression when delivered as an integral part of routine psychiatric care, which is consistent with our study. Additionally, our results indicate that individuals in the ICBT group experienced a more significant reduction of anxiety symptoms and nonspecific psychological distress than individuals in the WLC group. Previous studies showed that comorbidities of MDD and anxiety disorders were common, and the condition of patients would worsen with anxiety symptoms and psychological distress [64-66]. Therefore, it is very important for patients with MDD to improve anxiety symptoms and nonspecific psychological distress.

Notably, Shenzhen had a local outbreak of COVID-19, and several residential districts carried out strict lockdown measures during the later stages of our intervention. Some participants in the ICBT group expressed aggravation of depressive and anxiety symptoms owing to the outbreak and enforced isolation, and said that it was difficult to achieve self-regulation using CBT skills (such as behavioral activation and problem solving). Multiple studies reported that the COVID-19 pandemic had caused the deterioration of mental health [67-69], and the highest levels of depressive and anxiety symptoms occurred in the early stages of lockdown [70]. Thus, we assumed that this might have compromised the actual effect of ICBT in this study. However, the analysis results showed that there was no difference in depressive symptoms, anxiety symptoms, or nonspecific psychological distress between participants who had experienced the outbreak of COVID-19 and those who had not, whether in the ICBT group or WLC group. Thus, the local outbreak of COVID-19 did not affect our research results.

Meanwhile, we found that some participants in the ICBT group reported that they felt better, so they did not continue the modules or delayed the intervention until their symptoms worsened. Lundgren et al [20] speculated that incomplete treatment might have a negative impact on the effectiveness of ICBT, and that participants might improve more if they complete all modules. However, in our study, we compared all outcome measures between ICBT completers and noncompleters, and found no significant difference. The reason for this might be that most noncompleters dropped out of ICBT because they were feeling well and did not need ICBT to regulate their emotions. During the period from baseline to postassessment, the medication they received or the dissipation over time brought the emotions and symptoms of the ICBT noncompleters closer to those of the completers.

General self-efficacy refers to an individual’s self-confidence to deal with difficulties in daily life [71], and it affects the selection of coping strategies under challenging demands as the cognitive determinant of behavior [72]. Ludman et al [73] found that great self-efficacy was associated with self-management behaviors and improvements in depressive symptoms. Strong stigma affects patients’ correct understanding of their own diseases and help-seeking attitudes, reducing the use of mental health services [74,75]. We considered general self-efficacy and depression stigma as predictors of cognitive and behavioral changes in patients with MDD. The short-term relief of depressive symptoms only represents the treatment effect in the acute stage, but the changes in cognition and behavior may better influence the long-term prognosis of MDD. However, prior studies paid less attention to the impact of ICBT on self-efficacy and stigma. Our study included general self-efficacy and depression stigma as outcomes, which enriched the evaluation of the effect of ICBT in patients with MDD. The results indicated that our ICBT intervention could heighten participants’ general self-efficacy and might improve their help-seeking attitudes and treatment compliance, which was consistent with the results of the study by Topooco et al [60]. However, there was no significant difference in personal or perceived depression stigma between the groups. The initial goal of the ICBT development team was to create an atmosphere of social acceptance for patients with MDD by encouraging them to seek help from family and friends, and providing encouraging words during general support and course reminders, thereby relieving their personal and perceived depression stigma. However, the results of this study showed that the expected effect was not achieved. The reduction of stigma may require actual family involvement [76], more direct or indirect social contact, and long-term follow-up strategies [77].

The ultimate goal of treatment for MDD is to promote the recovery of patients’ social function and improve their HRQoL [14]. Our study results found no difference in social functional impairment or HRQoL between the ICBT group and the WLC group. Johansson et al [78] reported that nurse-delivered ICBT could reduce depressive symptoms and improve HRQoL in patients with cardiovascular disease. However, many separate studies have reported that ICBT had no evident impact on social functional impairment or HRQoL [20,61,79]. Generally, the effect of ICBT is mainly reflected in immediate emotional, cognitive, and behavioral changes. Yet, short-term psychotherapy cannot improve the level of social function or alleviate the physiological functional impairment and physical pain caused by MDD, so the improvement of social function and HRQoL by ICBT may take longer to reflect [20,80].

Our intervention was classified as unguided ICBT because we provided general support, but not support related to the therapeutic content. Notably, the adherence of ICBT in this study (31/40, 78%) is similar to the average adherence of guided ICBT (76%) and much better than that of unguided ICBT (54%) [29], and it has been reported that better adherence is correlated with a better treatment response [30]. Some participants said that the treatment content without face-to-face guidance was complicated and the homework was difficult, so they thought that ICBT was not suitable for them and they did not perceive great benefit from it. Additionally, some participants said that owing to their busy work or study schedules in daily life, they felt stress regarding the weekly modules, and that the task-based homework or videos increased their anxiety, so they delayed the modules or dropped out of the study. On the contrary, participants who felt that the modules were appropriate to their situations were more motivated to complete the modules and homework. Moreover, after receiving positive feedback on their symptom self-assessment, they felt that they had benefited from ICBT, so they rated it relatively highly and said that they would repeatedly complete the modules. Therefore, in future application and promotion, we should consider adding targeted guidance and feedback from professionals for people who have difficulty understanding the content of ICBT or those who are under severe psychological stress.

Many patients with mental disorders do not receive adequate treatment because of a lack of trained mental health specialists. It is necessary to apply treatments that are easy to implement in order to address the significant public health burden of depression. Nonspecialist health workers may therefore have an important role in delivering mental health care, and this may be a key strategy for closing the treatment gap [34]. Our intervention was implemented and supported by nonspecialists to achieve a higher adherence of ICBT, and it offered scalability through reduced reliance on mental health specialists.

On the other hand, most existing ICBT platforms are presented in the form of web pages (Mood GYM [28], E-Couch [81], etc) or computer programs (Beating The Blues [82], SPARX [83], etc), and the convenience of implementing ICBT interventions needs to be further improved. Behavioral inhibition and lack of motivation are two of the main clinical manifestations of patients with MDD. If the ICBT program is cumbersome to operate, patients might develop a mentality of avoidance or rejection, leading to treatment refusal or dropout. WeChat has become one of the most widely used social apps in China, and WeChat mini-programs have also become the main intelligent apps used by people in China. Compared with mobile apps, computer programs, and web pages, WeChat mini-programs do not need to be downloaded and installed, making them more convenient to use. Our study chose to implement the ICBT intervention using WeChat mini-programs as a platform, making the implementation of ICBT more convenient. With this approach, patients can participate in the ICBT intervention anytime and anywhere through their mobile phones. Furthermore, programs developed in Chinese may be more suitable for Chinese language habits, making it easier to accept ICBT treatment content. In this study, the majority of ICBT completers reported that they perceived moderate to enormous benefits and felt satisfied. Taken together, our ICBT program had great acceptability and satisfaction, and the findings may inform the implementation of other ICBT programs for MDD that increase access and adherence to treatment.

### Limitations

There are several limitations in this study. First, recruitment was only conducted in 2 hospitals in Shenzhen, and the majority of participants were women, were young individuals, and had high levels of education, which might limit the generalizability of the findings. On the one hand, the prevalence of MDD has been reported to be higher among women [6]. On the other hand, patients who were women and had high levels of education were more likely to visit health care services for treatment [84]. Our intervention and questionnaires were internet-based, so younger patients were more likely to take part in this study. Second, all outcomes were assessed using online self-report measurements. Generally, self-report measures are more conservative, and clinician-rated measures are more sensitive to improvement following psychotherapy for depression [85]. This may have caused measurement bias in this study. Third, our trial used a nonblind design owing to the nature of the intervention, which might have exaggerated the benefits generated by the intervention. Fourth, our study only involved results of the posttreatment assessment, and we did not collect and analyze follow-up data. Fifth, the recommended course of CBT for acute treatment is usually 12 to 16 weeks [14]. However, the period of ICBT in this study was only 7 weeks, which might be too short for patients with MDD to improve their mental health. Future studies should expand the research population, use a blind design, and explore the mid- and long-term effects of ICBT. Future researchers could adapt the content of ICBT interventions and extend the period of ICBT to achieve long-term and more pronounced relief of depressive symptoms while ensuring compliance.

### Conclusions

ICBT with general support delivered in this study showed greater short-term improvements in depressive symptoms, anxiety symptoms, nonspecific psychological distress, and general self-efficacy among select patients with MDD in comparison with the findings in waiting-list controls. It may be more suitable for implementation among people familiar with mobile phone and internet usage. Moreover, participants reported acceptability and satisfaction with our ICBT program, as evidenced by treatment adherence and participants’ feelings. Taken together, our results support the implementation of ICBT as an effective and scalable treatment option that increases access to mental health services among eligible people affected by MDD.

## Appendix

Multimedia Appendix 1 Outline of the treatment content and homework of the internet-based cognitive behavioral therapy program.

Multimedia Appendix 2 Household dysfunction questionnaire.

Multimedia Appendix 3 List of adverse life events.

Multimedia Appendix 4 Questionnaire to assess participants’ acceptability of and satisfaction with the internet-based cognitive behavioral therapy program.

Multimedia Appendix 5 Adjusted emotional symptom scores of participants who had experienced the outbreak of COVID-19 and those who had not at the posttreatment assessment in the study groups.

Multimedia Appendix 6 CONSORT-EHEALTH checklist (V 1.6.1).

## Abbreviations

CBT: cognitive behavioral therapy
FAS: full analysis set
GAD-7: Generalized Anxiety Disorder-7 Scale
GSES: General Self-Efficacy Scale
HRQoL: health-related quality of life
ICBT: internet-based cognitive behavioral therapy
K-10: Kessler 10-item Psychological Distress scale
MDD: major depressive disorder
MINI: Mini-International Neuropsychiatric Interview
PHQ-9: Patient Health Questionnaire-9
PPS: per-protocol set
RCT: randomized controlled trial
SDS: Sheehan Disability Scale
SF-6D: Short Form 6-Dimension
WLC: waiting-list control

## Data Availability

The data sets generated or analyzed during this study are not publicly available due to ethical restrictions but are available from the corresponding author on reasonable request.
