# Supplementary material for: The Effect of Internet-Based Cognitive Behavioral Therapy on Major Depressive Disorder: Randomized Controlled Trial
Source: J Med Internet Res. 2023 Sep 22;25:e42786. doi: 10.2196/42786 (PMC10559190; doi:10.2196/42786)
Supplement: Multimedia Appendix 2 [file jmir_v25i1e42786_app2.docx]

**Multimedia Appendix 2.** Household dysfunction questionnaire.

Did you have any of the following experiences in your childhood (before the age of 16)?

| Experiences | Yes | No |
| --- | --- | --- |
|  |  |  |
| Parents who had divorced. | 1 | 2 |
| Father or mother in prison. | 1 | 2 |
| Had witnessed or experienced domestic violence. | 1 | 2 |
| Lived with someone with mental disorder or suicide attempt. | 1 | 2 |
| Lived with someone with alcohol dependence or substance use disorder. | 1 | 2 |
| Economic difficulties. | 1 | 2 |
| Death of one or both parents. | 1 | 2 |
| Had witnessed or experienced neighborhood violence. | 1 | 2 |
| Discriminated against or treated unfairly on the grounds of gender. | 1 | 2 |
