# Supplementary material for: The Effect of Internet-Based Cognitive Behavioral Therapy on Major Depressive Disorder: Randomized Controlled Trial
Source: J Med Internet Res. 2023 Sep 22;25:e42786. doi: 10.2196/42786 (PMC10559190; doi:10.2196/42786)
Supplement: Multimedia Appendix 3 [file jmir_v25i1e42786_app3.docx]

**Multimedia Appendix 3.** List of adverse life events.

Did you have any of the following experiences in the last 6 months?

| Experiences | Yes | No |
| --- | --- | --- |
|  |  |  |
| You yourself suffered a serious illness, injury, or an assault. | 1 | 2 |
| A serious illness, injury, or assault happened to a close relative. | 1 | 2 |
| Your parent, child, or spouse died. | 1 | 2 |
| A close family friend or another relative(aunt, cousin, grandparent) died. | 1 | 2 |
| You had a separation due to marital difficulties. | 1 | 2 |
| You broke off a steady relationship. | 1 | 2 |
| You had a serious problem with a close friend.neighbor, or relative. | 1 | 2 |
| You became unemployed or you were seeking work unsuccessfully for more than one month. | 1 | 2 |
| You were sacked from your job | 1 | 2 |
| You had a major financial crisis. | 1 | 2 |
| You had problems with the police and a court appearance. | 1 | 2 |
| Something you valued was lost or stolen. | 1 | 2 |
| Child failed in further education or employment. | 1 | 2 |
| Difficulties in child discipline. | 1 | 2 |
| Child away from home for a long time. | 1 | 2 |
| High levels of stress at work or study. | 1 | 2 |
