# Supplementary material for: The Effect of Internet-Based Cognitive Behavioral Therapy on Major Depressive Disorder: Randomized Controlled Trial
Source: J Med Internet Res. 2023 Sep 22;25:e42786. doi: 10.2196/42786 (PMC10559190; doi:10.2196/42786)
Supplement: Multimedia Appendix 4 [file jmir_v25i1e42786_app4.docx]

**Multimedia Appendix 4.** Questionnaire to assess participants’ acceptability of and satisfaction with the internet-based cognitive behavioral therapy program.

- How many modules do you think you have actually completed seriously?

1 = Almost none, 2 = Fraction, 3 = Half, 4 = Majority, 5 = All.

- How much benefit do you think you have perceived from the courses in this programme?

1 = None at all, 2 = A little, 3 = Moderate, 4 = Great, 5 = Enormous.

- Are you satisfied with the program's curriculum?

1 = Very dissatisfied, 2 = Dissatisfied, 3 = Average, 4 = Satisfied, 5 = Very satisfied.

- Would you like to recommend this ICBT program to your family or friends with depressive symptoms?

1 = Yes, 2 = No.
