# Supplementary material for: The Effect of Internet-Based Cognitive Behavioral Therapy on Major Depressive Disorder: Randomized Controlled Trial
Source: J Med Internet Res. 2023 Sep 22;25:e42786. doi: 10.2196/42786 (PMC10559190; doi:10.2196/42786)
Supplement: Multimedia Appendix 5 [file jmir_v25i1e42786_app5.docx]

**Multimedia Appendix 5.** Adjusted emotional symptom scores of participants who had experienced the outbreak of COVID-19 and those who had not at the posttreatment assessment in the study groups.

| Group | Outcome measure | Participants who had experienced the outbreak^a^ | Participants who had not experienced the outbreak^a^ | *F* | *P* value |
| --- | --- | --- | --- | --- | --- |
|  |  |  |  |  |  |
| ICBT^b^ group | PHQ-9^c^ | 5.89 (1.03) | 6.94 (1.62) | 0.295 | .59 |
|  | GAD-7^d^ | 5.18 (0.94) | 5.67 (1.49) | 0.073 | .79 |
|  | K-10^e^ | 20.32 (2.29) | 21.88 (3.59) | 0.132 | .72 |
| WLC^f^ group | PHQ-9 | 11.53 (2.20) | 10.80 (0.80) | 0.099 | .76 |
|  | GAD-7 | 8.51 (2.06) | 7.67 (0.75) | 0.147 | .70 |
|  | K-10 | 30.37 (2.92) | 25.90 (1.06) | 2.068 | .16 |

^a^Adjusted mean (SD) score of each outcome measure at post-treatment assessment using covariance analysis with the respective baseline outcome measure score as a covariate.

^b^ICBT: internet-based cognitive behavioral therapy.

^c^PHQ-9: Patient Health Questionnaire-9.

^d^GAD-7: Generalized Anxiety Disorder-7 Scale.

^e^K-10: Kessler 10-item Psychological Distress scale.

^f^WLC: waiting-list control.
